# Overall equipment effectiveness, efficiency and slide review analysis of high-end hematology analyzers

**DOI:** 10.1016/j.plabm.2022.e00275

**Published:** 2022-04-18

**Authors:** Shubham Rastogi, Sukesh Chandran Nair, Pandiyan Murugan, Asady Sukanya Sukumar, Joy J. Mammen, Saravanan Mullai

**Affiliations:** a Department of Transfusion Medicine & Immunohematology, Christian Medical College, Vellore, Tamil Nadu, India; b HORIBA Medical, HORIBA ABX SAS, Parc Euromédecine - Rue du Caducée, France

**Keywords:** Overall equipment effectiveness, High-end hematology analyzers, Yumizen H2500, Sensitivity, Specificity, Efficiency

## Abstract

This study evaluated and compared the overall equipment effectiveness (OEE), sensitivity, specificity, and efficiency of the high-end hematology analyzers, Yumizen H2500, DxH 800, DxH 900 and XN-9000 (XN-10). A total of 400 anonymized left over’s K2 EDTA whole blood samples were analyzed for complete blood count. Of 400 samples, 200 were tested on Yumizen H2500; DxH 800 & DxH 900 while the other 200 were tested on Yumizen H2500 & XN-9000 (XN-10), respectively. The OEE was good and comparable for all the hematology analyzers except DxH 800 showing an average status. The sensitivity (%), specificity (%) and turnaround time (in minutes) for Yumizen H2500, DxH 800, DxH 900 and XN-9000 (XN-10) were 91.67, 61.11 & 103; 66.67, 54.55, & 149; 83.33, 27.27 & 136; 83.33, 28.57 & 122, respectively. Confusion matrix highlights the difficulty for DxH 800 and DxH 900 to discriminate left shift or blasts with large hyper-segmented neutrophils. The flags triggered by Yumizen H2500 were markedly changed to large hyper-segmented neutrophils. Lymphoblast caused more confusion for XN-9000 (XN-10), as it came out to be atypical lymphocytes, or hypersegmented neutrophils. Although comparable in OEE index to other analyzers, the Yumizen H2500 seems to be more reliable in detecting the abnormal cells as it has high sensitivity, specificity and less turnaround time. Thus, analysis adding specificity, sensitivity, and efficiency parameters to the OEE index provides more reliable information of the analyzers.

## 1 Introduction

In clinical laboratories with a heavy patient load, the testing quality of the automated hematology analyzers depends on their accuracy, reliability, and performance as well as efficiency. Continuous evaluation of the equipment with the appropriate methods is the key to ensure sustained quality of reporting in clinical laboratories with a heavy patient load. However, the approaches used for maintaining quality control (QC) include equipment calibration every 6 months, the daily measurement of QC standards, and the inter-instrument comparison within the 6-month interval using patient samples (>40 samples) [1,2].

Although the performance of the automated hematology analyzers expressed as turn-around time is one of the important criteria in high-volume testing labs to deliver patient quality results on time, it may not be comparable for the analyzers from different companies. Therefore, these analyzers should be compared and evaluated in real-time, using overall equipment effectiveness (OEE) method [3]. The metrics behind OEE utilizes three factors such as availability, performance and quality to measure the percentage of the truly productive time [4].

Only few studies have compared different hematology analyzers in parallel [[5], [6], [7], [8]]. None of them has compared the OEE for different hematology analyzers as the definition of OEE does not include all variables that decrease the capacity utilization of clinical laboratory equipment. Arguments are made that sensitivity, specificity, and efficiency should usually be applied in the context of comparing the clinical equipment used for the patient's specimen. Therefore, technology that reduced interference or flagging rates by increasing specificity and sensitivity of equipments have the potential to significantly improve workload and turn-around times without endangering patients by reporting false or misleading results. Thus, the objective of the present study is to evaluate and compare the OEE, sensitivity, specificity, and efficiency for four contemporary high-end hematology analyzers in the setting of a large tertiary care hospital from Southern India.

## 2 Materials and methods

### 2.1 Study design, center and hematology analyzers

This was a prospective cross-sectional comparative study of 4 hematology analyzers [Yumizen H2500 (Horiba, Kyoto, Japan), the DxH 800 and DxH 900 (Beckman Coulter, Miami, USA), and the XN-9000 (XN-10) (Sysmex Corporation, Japan)] available as a routine instrument in the central laboratory of Christian Medical College, Vellore, India. As of 2019, the center has the capacity to process 3500–4000 samples per day of various pathologies. Each hematology analyzer had its maintenance record having all pertinent information, which made it possible to assess the condition of equipment and perform necessary maintenance.

The Yumizen H2500 (Horiba, Kyoto, Japan) uses the double hydrodynamic sequential system and the impedance method. The Yumizen H2500 mixes the samples at 360° to ensure their homogeneity. The analyzer capacity is 120 samples per hour and the sample volume needed for analysis is 110 μl [9].

Both DxH 800 and DxH 900 use impedance, and five light scatter and absorption measurements (volume, conductivity, and scatter [VCS] technology) method for counting white blood cells (WBCs) differential, red blood cells (RBCs), platelets (PLT), and nucleated red blood cells (NRBC). The VCS technology allows improved data acquisition per sample. The capacity is twenty separate five-tube cassettes with a maximal automated throughput of 100 samples per hour [[10], [11], [12]].

This Sysmex XN-10 uses fluorescence and flow cytometry technology with a semiconductor laser to categorize WBCs. In case of additional fluorescence interference, it uses a recently developed pRBC flag to correct WBC counts on the WBC differential scattergram [13].

### 2.2 K2 EDTA whole blood samples

To permit unbiased testing of the four instruments, a total of 400 anonymized left over’s K2 EDTA whole blood samples, out of routine diagnostics and without prior knowledge of blood count analysis or clinical background, were collected in 2 ml tube, Samples with inadequate quantity, inappropriate blood to anticoagulant proportion, or tiny clots were excluded from the study.

### 2.3 Specimen analysis

Under aseptic precautions, whole blood samples were mixed well by gentle inversion. CBC analysis of all blood samples was performed using the different automated hematology analyzers. Of 400 samples, 200 were processed on Yumizen H2500; DxH 800 & DxH 900 (Set 1) while the other 200 were processed on Yumizen H2500 & XN-9000 (XN-10) (Set 2), respectively. During the entire period of study, the quality assurance or quality control procedures were followed.

#### 2.3.1 Criteria for flagging

Each sample was reviewed according to the laboratory auto-validation criteria for hematology analyzers. If a specified flag appeared and/or output was beyond the specified range, a rule in the criteria would be triggered. Thin blood smears were prepared for all the flagged samples and were stained with Leishman stain. Manual peripheral smear review was performed to identify morphological abnormalities, immature cells and to confirm results produced by the analyzer.

### 2.4 Comparative assessment of equipment

The comparisons of flagging quality, manual microscopy and inter-instrument efficiency were done as per Clinical and Laboratory Standards Institute (CLSI) guidelines [1]. Blood smear analysis was done by two experts, performing a 200-cell manual differential. Left shift was defined as a band count ≥8% in blood smear.

### 2.5 Statistical analysis

All statistical analyses were performed using Microsoft Excel software (Microsoft Corp., Redmond, WA).

#### 2.5.1 Overall equipment effectiveness

The OEE was calculated for each hematology analyzer using the following formula.

Overall equipment effectiveness (OEE) = Availability × Performance × Quality

According to the OEE index, 100% effectiveness is an ideal and only theoretical performance. However, analyzers that are above 90% are more effective and come under the good category. 80–90% of OEE analyzers are defined as the average OEE category.

##### 2.5.1.1 Availability

Availability is the ratio of the runtime to the planned testing time. It takes into consideration the availability losses. Availability losses are all the downtimes that the process faces during the time that it is supposed to be running. Also, there are planned stops that may occur because of changeover and unplanned stops that may be caused by lack of samples or equipment failure, and then the remaining time from the whole testing time, deducting the availability loss, is called the run time. The availability is calculated by the following equation:

Availability = Runtime/Planned testing time

##### 2.5.1.2 Performance

Performance is the ratio of the net run time to the practical run time, and this factor takes into consideration anything that may reduce maximum speed of testing, including minor stops and slow cycles. Mathematical calculation of the performance is done by the following equation:

Performance = Ideal one CBC time × Total CBC count/Practical run time.

Where the ideal one CBC time is the theoretical time to perform one CBC claimed by the manufacturer, total CBC count is the total number of samples performed by the hematology analyzer without consideration of quality and practical run time is the period on the hematology analyzers is required for an operator to perform total counts.

##### 2.5.1.3 Quality

The quality factor takes into consideration that whether or not all the results that occurred during the testing process met the quality standards. To calculate the quality the following equation was used:

Quality: auto-validated count/total CBC count

Where the auto-validated count is the number of the results that met the quality standards (reference) and the total is the number of all CBC count. A sample was classified as true positive (TP) if it was selected to review by certain screening criteria (SC) and the microscopic analysis produced some positive smear findings (PSF). A sample was classified as false positive (FP) if it was selected to review by SC with no PSF in microscopy. A sample was classified as true negative (TN) if it was not selected to review by any SC and the manual blood smear review (MBSR) did not show any PSF. Finally, a sample was classified as false negative (FN) if it was not selected to review by any SC and the MBSR contained some PSF. To compare the hematology analyzers, a confusion matrix was calculated for slide reviews triggered by flag. The accuracy, sensitivity, specificity, precision, and F1-Measure of the analyzers were also calculated using the following equations [14].

Accuracy (%) = (TP + TN)/TP + FP + TN + FN) x 100

Sensitivity (%) = TP/ (TP + FN) x 100

Specificity (%) = TN/ (TN + FP) x 100

Precision (%) = TP/ (TP + FP) x 100

F1-measures (%) = 2 x (Sensitivity x precision)/ (Sensitivity + Precision)

## 3 Results

### 3.1 Overall equipment effectiveness

**Fig. 1:**
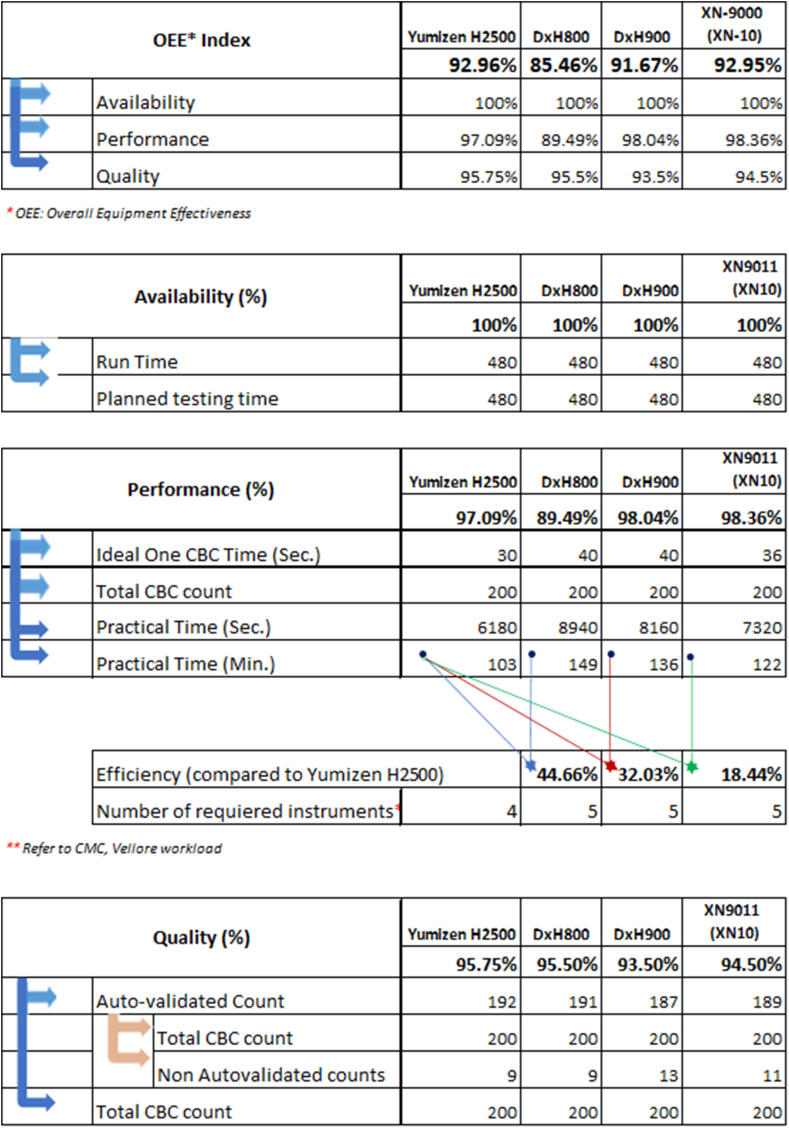
Overall equipment effectiveness of 4 hematology analyzers.

The OEE of Yumizen H2500, the DxH 800 and DxH 900, and the XN-9000 (XN-10) were 92.96%, 85.46%, 91.67%, and 92.95%, respectively. OEE was observed to be good with the Yumizen H2500, DxH 900, and XN-9000 (XN-10), while the DxH 800 showed its average value based on the established OEE index. The availability was 100% for all the instruments, as there was no breakdown and instruments worked well during the analysis as shown in Fig. 1.

### 3.2 Performance of hematology analyzers

The performance, as measured by total CBC count, of Yumizen H2500, DxH 800, DxH 900 and XN-9000 (XN-10) was 97.09%, 89.49%, 98.04% and 98.36% respectively. Of the 200 samples processed on different hematology analyzers, the total number of flags generated by Yumizen H2500, DxH 800, and DxH 900 were 9, 9, and 13 respectively. Similarly, 200 samples were processed on Yumizen H2500 and XN-9000 (XN-10). Total flags generated by the Yumizen H2500 and XN-9000 (XN-10) were 8 and 11 respectively.

The Yumizen H2500 required approximately 103 min for total 200 samples analysis in comparison to 149, 136, and 122 min for DxH 800, DxH 900 & XN-9000 (XN-10) hematology analyzers, respectively. This time was analyzed on practical laboratory scenario where samples were selected randomly. The DxH 800, DxH 900, and XN-9000 (XN-10) analyzers were about 44.66%, 32.03%, and 18.44% less efficient compared with Yumizen H2500.

### 3.3 Output quality

**Fig. 2:**
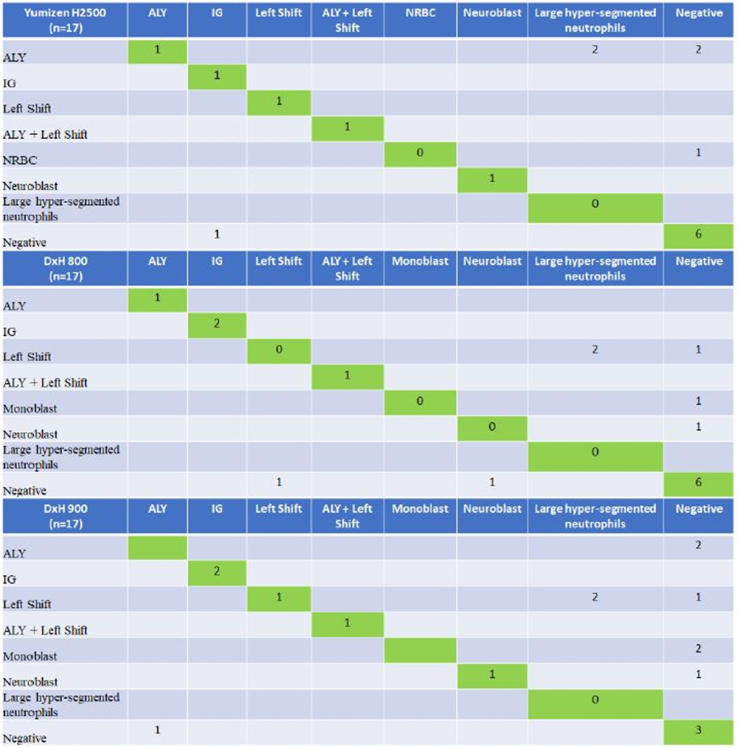
Confusion matrix of Yumizen H2500, DxH 800, and DxH 900 hematology analyzers (ALY – Atypical Lymphocytes, IG – Immature Granulocytes, NRBC – Nucleated Red Blood Cells). (For interpretation of the references to colour in this figure legend, the reader is referred to the Web version of this article.)

**Fig. 3:**
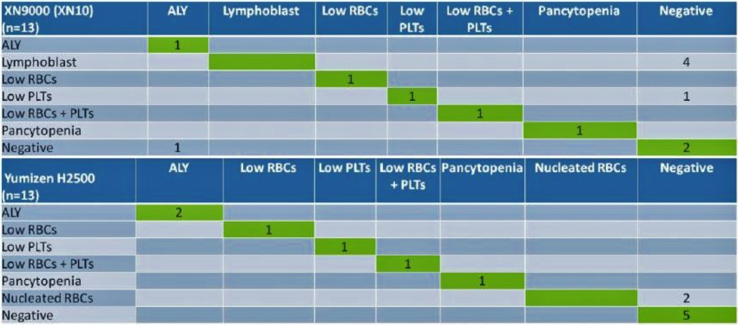
Confusion matrix of Yumizen H2500 and XN-9000 (XN-10) hematology analyzers (ALY – Atypical Lymphocytes, RBC – Red Blood Cells, PLTs – Platelets). (For interpretation of the references to colour in this figure legend, the reader is referred to the Web version of this article.)

The output quality of Yumizen H2500, DxH 800, DxH 900 and XN-9000 (XN-10) was 95.75%, 95.50%, 93.50% and 94.50%, respectively. The slides of flagged samples by all analyzers were prepared and verified with the expert microscopic cell counts. False-negative output on each of the instruments (Yumizen H2500, DxH 800, DxH 900) was comparable, but false-positive output for Yumizen H2500 was lower than others (Table 2, Table 3). This has important implications for slide review rates. In set 1, the DxH 900 had the highest false-positive rate [4% (8/200)], followed by the DxH 800 [2.5% (5/200)] and the Yumizen H2500 [2.5% (5/200)]. The rates of false-positive results in set 2 were 1% (2/200) and 2.5% (5/200) for Yumizen H2500 analyzer and XN-9000 (XN-10), respectively. Table 1 shows the accuracy, sensitivity, specificity, precision, and F1-Measure of Yumizen H2500, DxH 800, DxH 900 and XN-9000 (XN-10) analyzers respectively. Fig. 2 presents the confusion matrix for Yumizen H2500, DxH 800, and DxH 900 hematology analyzers. Left shift/blasts and large hyper-segmented neutrophils cause more confusion for DxH 800, and DxH 900; 6 samples (3 on DxH 800 and 3 on DxH 900) of left shift and 4 samples of blasts (2 on DxH 800 and 2 on DxH 900) were classified as large hyper-segmented neutrophils. Fig. 3 presents the confusion matrix for Yumizen H2500, and XN-9000 (XN-10) hematology analyzers. Lymphoblast/NRBC and negative results cause more confusion. 4 samples of lymphoblast on XN-9000 (XN-10) were classified as negative, and 2 samples of NRBC on Yumizen H2500 were classified as negative.

**Table 1:** Comparison between Yumizen H2500, DxH 800, DxH 900 and XN-9000 (XN-10) hematology analyzers.

Parameters	Yumizen H2500 (Aggregate)	DxH 800 (Set 1)	DxH 900 (Set 1)	Yumizen H2500 (Set 1)	XN-9000 (XN-10) (Set 2)	Yumizen H2500 (Set 2)
Accuracy, (%)	73.33	58.82	47.06	64.71	53.85	84.62
Sensitivity, (%)	91.67	66.67	83.33	83.33	83.33	100.00
Specificity, (%)	61.11	54.55	27.27	54.55	28.57	71.43
Precision, (%)	61.11	44.44	38.46	50.00	50.00	75.00
F1-measure, (%)	73.04	52.80	52.13	62.41	62.41	85.71
True positive, (n)	11	4	5	5	5	6
True negative, (n)	11	6	3	6	2	5
False positive, (n)	7	5	8	5	5	2
False negative, (n)	1	2	1	1	1	0

**Table 2:** Comparison regarding reason for non-autovalidation between Yumizen H2500, DxH 800 and DxH 900 hematology analyzers (n = 17).

Yumizen H2500	Conclusion	DxH 800	Conclusion	DxH 900	Conclusion	Slide review comments
–	True Negative	NE blast	False Positive	NE blast	False Positive	Large hypersegmented neutrophils falling in flag zone.
–	True Negative	Monoblast	False positive	Monoblast	False positive	Large hypersegmented neutrophils falling in flag zone.
ALY	False positive	Left shift	False positive	Left shift	False positive	Large Monocytes falling in flag zone Some of the large neutrophils with hypersegmentation overlapped the ALY flag system
	False Negative	IG	True positive	IG	True positive	Higher proportion of IG was present in blood smear
ALY	True positive	ALY	True positive	–	False negative	Monocytosis with reactive & large lymphocytes causing the ALY flag
ALY + Left shift	True positive	ALY + Left shift	True positive	ALY + Left shift	True positive	Lymphoid crisis, ALY, Band count ≥8% in blood smear
IG	True Positive	IG	True positive	IG	True positive	Higher proportion of IG was present in blood smear
ALY	False positive	Left shift	False positive	Left shift	False positive	Large neutrophils with hypersegmentation falling in flagging zone and overlapping into ALY/LY
–	True Negative	Left shift	False positive	Left shift	False positive	Large hypersegmented neutrophils falling in IG flag zone
ALY	False Positive	–	True negative	–	True negative	Large neutrophils with hypersegmentation falling in flagging zone and overlapping into ALY/LY
NRBC	False Positive	–	True negative	–	True negative	Negative for NRBC
ALY	False Positive	–	True negative	–	True negative	Large neutrophils with hypersegmentation falling in flagging zone and overlapping into ALY/LY
NE blast	True Positive	–	False negative	NE blast	True positive	Positive for NE blast
–	True Negative	–	True negative	ALY	False positive	Large neutrophils with hypersegmentation falling in flagging zone and overlapping into ALY/LY
–	True Negative	–	True negative	ALY	False positive	Large neutrophils with hypersegmentation falling in flagging zone and overlapping into ALY/LY
–	True Negative	–	True negative	Monoblast	False positive	Large hypersegmented neutrophils falling in flag zone.
Left shift	True Positive	–	False negative	Left shift	True positive	Band count ≥8% in blood smear

Abbreviations: ALY- Atypical Lymphocytes, IG – Immature Granulocytes, NRBC – Nucleated Red Blood Cells, NE blast – Neutroblast; CML – Chronic myeloid leukemia.

**Table 3:** Comparison regarding reason for non-autovalidation between Yumizen H2500 and XN-9000 (XN-10) hematology analyzers (n = 13).

S. No	Yumizen H2500	Conclusion for slide	XN-9000 (XN-10)	Conclusion for slide	Slide review comments
1		True negative	Lymphoblast	False Positive	Occasional reactive lymphocytes seen which agrees with ALY but negative for lymphoblast.
2		True negative	Low PLTs	False Positive	Slide review came because MPV was not reported in XN in case of thrombocytopenia but in Yumizen H2500, MPV was reported.
3	Pancytopenia	True Positive	Pancytopenia	True Positive	Positive for pancytopenia
4	Low RBCs/Low platelets	True Positive	Low RBCs/Low platelets	True Positive	Positive for low RBCs/low platelets
5	Low RBCs	True Positive	Low RBCs	True Positive	Positive for low RBCs
6	ALY	True Positive	ALY	True Positive	Positive for ALY
7	Low Platelets	True Positive	Low Platelets	True Positive	Slide review showed low platelet count
8		True negative	Lymphoblast	False Positive	Negative for lymphoblasts
9		True negative	Lymphoblast	False Positive	Smear showed smudge cells. These could be reactive lymphocyte and large hypersegmented neutrophils.
10		True negative	Lymphoblast	False Positive	Proportion of ALY is less. But Slide reveals hypogranular or pseudo-Pelger-Huët neutrophils
11	NRBC	False Positive	Slide was not required	True negative	Negative for NRBC
12	ALY	True Positive		False negative	Positive for ALY. More smudge cells
13	NRBC	False Positive	PLT aggregates	True negative	NRBC were not there but PLT aggregates and Large PLT were seen.

Abbreviations: ALY- Atypical Lymphocytes, IG – Immature Granulocytes, NRBC – Nucleated Red Blood Cells, PLT – Platelets, MPV – Mean Platelet Volume.

## 4 Discussion

Timely and accurate reporting of blood cell count and differentials is the primary goal of clinical hematology laboratories. The key factors that influence hematology analyzer selection include reliability of the analyzer, cost of analyzer, availability, and turnaround time. Since manual verification of the obtained result is labor-intensive and time-consuming, there is a need to use a more accurate and sensitive hematology analyzer to effectively handle many samples with minimum mistakes and slide review. The currently used inter-instrument comparison approach includes a periodic regression analysis that uses at least 40 CBC samples of patients [1,2]. When the laboratory uses this strategy, the consistency of multiple instruments cannot be confirmed. Hence, in this study the performance, maintenance, and reporting quality of 4 different hematology analyzers were compared using OEE method and the reliability was assessed using manual microscopy as the gold standard. In this study, turnaround time was also measured as an indicator of performance because this improved the workflow rate in large volume laboratories.

In this study, the OEE of different automated analyzers was compared in terms of the total number of CBC parameters performed at a particular point within the shift, day, or production run. The OEE was good and comparable for all the hematology analyzers except DxH 800 showing an average status. A study by Chabert et al. comparing the performance of Yumizen H2500 hematology analyzer to other blood counter models in terms of precision and linearity reported that the Yumizen H1500 and H2500 instruments are as safe, as effective, and perform as well or better than the Advia 2120, Sysmex XE2100 and XN10 instruments for all the usual CBC parameters [15].

Cellular interference may influence the WBC, PLT, and NRBC counts that lead not only reduction in operational efficiency of the hematology laboratory, but also inaccurate clinical decision making [[16], [17], [18], [19], [20], [21], [22]]. The presence of interfering particles in automated blood cell enumeration often triggers a ‘flag’ for manual review. A ‘flag’ from the hematology analyzer is a notification that a reported result might be incorrect. This means that either a result is outside the established normal ranges programmed into the instrument (resulting in false negatives) or the hematology analyzer misidentifies other cellular types (resulting in false positives) [[23], [24], [25], [26], [27]]. In 20%–25% of CBCs, abnormal cell flags are generated, which needs manual film reviews [28,29]. However, manual film reviews range varies from 10% to 50% in different laboratories depending on the local guidelines and clinical population [29,30]. In this study, the manual blood smear review of all blood samples that cause flagging was performed by two experts for increasing the results credibility. Two levels were evaluated: the first level was to check the presence of a flag (independently of its accuracy) and the second level was to check the accuracy of the flag.

In 2016, Eldanasoury et al. reported that the suspect flags were accountable for 60.2% of their false-positive results [31]. They indicated that the hematology analyzers were responsible for an increase in unnecessary manual smear review due to over flagging. Similarly, several other studies have also indicated higher number of manual film reviews triggered by abnormal cell flags [14,31,32]. In contrast, our findings indicate lower number (about 4%–7%) of manual film reviews for each of the analyzers triggered by abnormal cell flags. Kim et al. stated that the slide review rate might vary among the different analyzers studied and that only most appropriate analyzers should be selected by individual laboratories based on clinical characteristics such as clinic size and patient population [33]. In this study, the efficiency evaluation of all the four hematology analyzers was obtained by assessment of the turnaround time for complete blood count in the laboratory. Our findings indicate that the use of the Yumizen H2500 in the routine laboratory would reduce the turnaround time from about 149 min, 136 min, and 122 min on the DxH 800, DxH 900, and XN-9000 (XN-10), to about 103 min. With a daily workload of about 3500 samples in CMC Vellore, this difference represents a reduction of about one Yumizen H2500 (from about 5 instruments to 4 in numbers). The low turnaround time and number [about 4% (17/400)] of manual film reviews for Yumizen H2500 highlights the importance of ongoing software appraisal and optimization.

Studies have shown highly sensitivity, specificity and accuracy of flags on DxH 800 [11,34,35]. However, the high flagging sensitivity for the XN series and markedly lower sensitivity for the DxH 800 have also been indicated in other studies [7,8,36]. Our findings indicate high flagging sensitivity for the Yumizen H2500, DxH 900 and XN-9000 (XN-10) compared with DxH 800. Beside high flagging sensitivity, low false-positive alerts and high specificity are the other important parameters to reduce unnecessary manual smear reviews. Studies have shown that the flagging parameters most contributing to the number of false-positive samples were IGs followed by left shift and PLT clumping [[5], [6], [7],^33^].

Combining flagging alerts for presence of blasts, left shift and atypical/abnormal lymphocytes, DxH 900 revealed the lowest specificity (27.27%) among all investigated instruments in this study primarily due to a relatively high number of false-positive blasts and left shift warnings. In this study, XN-9000 (XN-10) was the second instrument with less specificity (28.57%) after the DxH 900 due to a relatively high number of false-positive monoblasts. In contrast, a previous study by Jones et al. comparing XN-1000 with XE-500 for sensitivity and specificity of flagging for abnormal WBC on pediatric samples reported XN-1000 superiority over XE-5000, with greater reduction in blood films for review [37]. However, the flag specificity for blood samples collected from infants between 8 days and 2 years of age on XN-1000 was <35%, which increased up to 67% thereafter [38]. Yumizen H2500 analyzer with low false positive rate in this study exhibited a high flagging sensitivity (91.67%) and specificity (61.11%). The high sensitivity, precision and specificity of Yumizen H2500 can be explained owing to improved cell separation (360°), ongoing software appraisal and optimization. This results in significant decrease in unnecessary morphology reviewing by microscopy, thus saving significant time in the laboratory.

Confusion matrix (Fig. 2) highlights the difficulty for DxH 800 and DxH 900 to discriminate left shift or blasts with large hyper-segmented neutrophils. The negative flags triggered by Yumizen H2500 were markedly changed after manual slide review to large hyper-segmented neutrophils. Lymphoblast caused more confusion for XN-9000 (XN-10), as it came out to be atypical lymphocytes, hypersegmented neutrophils, or hypogranular neutrophils.

Overall, Yumizen H2500 appears to be a satisfactory analyzer for the common clinical laboratory use; though comparable to DxH 900 & XN-9000 (XN-10) hematology analyzers in terms of OEE index. This index measures machine effectiveness based on the availability and performance rate element to obtain the actual maintenance performance level, without considering problems related to reliability (specificity and sensitivity) and turnaround time (efficiency) parameters. Based on this study, the structured technique of the modified OEE index can be proposed [SnSr index named after author’s names Sukesh Nair (Sn) and Shubham Rastogi (Sr)]. This index uses specificity, sensitivity, and efficiency parameters along with the OEE index for identifying the usefulness of the laboratory instrumentations. According to SnSr index, 100% effectiveness, efficiency, sensitivity, and specificity are ideal and only theoretical performance. However, analyzers that are above 90% are more effective and come under the good category. 80–90% analyzers are defined as average on the SnSr index.

The authors want to highlight few limitations present in the study: (i) the sensitivity, specificity and efficiency analysis of Yumizen H2500 is based on blood smear evaluation of a small number of samples. (ii) In this study the observers were not blinded and had the access to reports of hematology analyzers (though not referred to), which might have resulted into morphological changes over-reporting, particularly those marked by suspect flags. (iii) The data presented are generated from a single population with a specific profile in a CMC Vellore that uses a certain type of hematology analyzer.

## 5 Conclusion

All the 4 hematology analyzers showed comparable OEE in technical evaluation. However, the Yumizen H2500 analyzer seems to be more reliable in detecting the abnormal cells as it has high sensitivity, specificity and less turnaround time compared to other analyzers. Thus, analysis adding specificity, sensitivity, and efficiency of the laboratory instrumentations to the OEE index provides more reliable information on accuracy, productive time and turnaround time.

## Author's contribution

SCN, SR, CF, JJM designed the study; SM, PM and ASS performed laboratory experiment, while SCN performed blood smear analysis and SR was involved in data compilation and analysis. All the authors approved the final version of the article submitted.

## Declaration of competing interest

Shubham Rastogi is the employee of HORIBA ABX SAS. SCN, JJM, SM, PM and ASS declare no conflict of interest.
