# Supplementary material for: Overall equipment effectiveness, efficiency and slide review analysis of high-end hematology analyzers
Source: Pract Lab Med. 2022 Apr 18;30:e00275. doi: 10.1016/j.plabm.2022.e00275 (PMC9117814; doi:10.1016/j.plabm.2022.e00275)
Supplement: Multimedia component 1 [file mmc1.pdf]

Slide Review Criteria

	Document Name	CMCTMIH – R 37
	Version	3.0

S#	Parameters	Rules	comments
1	Platelet	< 50000	slide review
2	Platelet	50000-100000 and MPV >10.5	no slide review
3	Haemoglobin	<6gm (>20gm/dL in case of neonates)	slide review
4	MCV	<60 >110	slide review
5	MCHC	>35.0	slide review
6	RDW	>25.0	slide review
7	RDW	Excludes OGpatient,dimorphic	
8	Retic	> 5% #±0.1	slide review
**Differential**			
9	Neutrophil	Any value	no slide review
10	Eosinophil	Any value	no slide review
11	Lymphocyte	Adult >50 child >60 till 15 yrs (No slide review if absolute number of lymphocytes#< 5000)	slide review
12	Monocyte	>15% (If histogram reflects monocytosis), Exclude slide review if patient on radiation and absolute number < 1000	slide review
13	NRBC	>2	slide review
**Suspect flags**			
14	Immature granulocytes (IG)/ Shift left	Only with NRBC's	slide review
15	Variant lymphocyte	If significant numbers	slide review
16	Ne blast		slide review
17	Mono blast		slide review
18	Lympho blast		slide review
19	Less than 500 cells	No differential (Do manual only for the first time, from the next time onward validate with a message **"DC not done"**)	

Technical review	Quality Review	Authorized By	Issued	Review
[signature]	[signature]	[signature]		
Section in charge	Quality Manager	HOD	5/15/2019	5/15/2019
