# Supplementary material for: Overall equipment effectiveness, efficiency and slide review analysis of high-end hematology analyzers
Source: Pract Lab Med. 2022 Apr 18;30:e00275. doi: 10.1016/j.plabm.2022.e00275 (PMC9117814; doi:10.1016/j.plabm.2022.e00275)
Supplement: Multimedia component 2 [file mmc2.pdf]

# Software version of all analyzers:

| | Analyzers | Vresion |
|---|---|---|
| a | Sysmex XN900(10) | 22.11-00 |
| b | Horiba YH2500 | V1.3.0 |
| c | DxH 800 | 3.9.0.31 |
| d | DxH 900 | 1.1.0.96 |
